# Biotransformation of American Ginseng Stems and Leaves by an Endophytic Fungus *Umbelopsis* sp. and Its Effect on Alzheimer’s Disease Control

**DOI:** 10.3390/nu15234878

**Published:** 2023-11-22

**Authors:** Qiqi Chen, Jingying Wang, Yuhang Gao, Zixin Wang, Xiujun Gao, Peisheng Yan

**Affiliations:** 1 School of Environment, Harbin Institute of Technology, Harbin 150090, China; 19b929072@stu.hit.edu.cn (Q.C.);; 2 School of Marine Science and Technology, Harbin Institute of Technology, Weihai 264209, China

**Keywords:** Alzheimer’s disease, American ginseng stems and leaves, biotransformation, endophytic fungus, solid-state fermentation

## Abstract

Background: Common ginsenosides can be transformed into rare ginsenosides through microbial fermentation, and some rare ginsenosides can prevent Alzheimer’s disease (AD). This study aimed to transform common ginsenosides into rare ginsenosides through solid-state fermentation of American ginseng stems and leaves (AGSL) by an endophytic fungus and to explore whether fermented saponin extracts prevent AD. Methods: The powders of AGSL were fermented in a solid state by endophytic fungus. Total saponins were extracted from fermentation products using the methanol extraction method. The types of saponins were analyzed by liquid chromatography mass spectrometry (LC/MS). The Aβ42 concentration and β-secretase activity were measured by ELISA for the prevention of AD. Results: After AGSL was fermented by an endophytic fungus NSJG, the total saponin concentration of the fermented extract G-SL was higher than the unfermented CK-SL. Rare ginsenoside Rh1 was newly produced and the yield of compound K (561.79%), Rh2 (77.48%), and F2 (40.89%) was increased in G-SL. G-SL had a higher inhibition rate on Aβ42 concentration (42.75%) and β-secretase activity (42.22%) than CK-SL, possibly because the rare ginsenoside Rh1, Rh2, F2, and compound K included in it have a strong inhibitory effect on AD. Conclusion: The fermented saponin extracts of AGSL show more inhibition effects on AD and may be promising therapeutic drugs or nutrients for AD.

## 1. Introduction

Ginsenosides are the main active ingredients of ginseng, which has a large source, rich content, high efficacy, a broad spectrum of biological activity, and a high curative effect [1,2]. According to research, ginsenosides have good healthcare functions for cardiovascular disease and nervous system disease [3,4,5]. They also have hypoglycemic [6,7], hypotensive [8], antitumor [9,10], antiaging [11], and antifatigue effects [12]. Generally, ginsenosides are converted into rare ginsenosides with short sugar chains in the body to exert biological activity. Rare ginsenosides have a lower polarity and are more easily absorbed into the blood by the body, so they have a higher medicinal value [13]. Sources of rare ginsenosides are limited due to their low content in wild plants, hindering their application to functional foods and medicines. Many studies have found that microbial fermentation can transform common saponins with high content into rare saponins to improve medical value [14]. Therefore, it is particularly important to transform common ginsenosides into rare ginsenosides to maximize the efficacy of Panax ginseng and American ginseng. Usually, ginsenosides are mainly obtained from the roots, but the stems, leaves, and other parts still contain active ingredients [15]. The cost of obtaining ginsenosides from stems and leaves is low, and transforming common ginsenosides from stems and leaves into rare ginsenosides can save costs, increase biological activities, and realize the comprehensive utilization of stems and leaves.

In recent years, there have been some scientific reports on endophytic fungi in Panax plants. Endophytic fungi are microorganisms that live in the intercellular spaces of plant tissues and can form temporary or long-term symbiotic relationships with plants [16]. Furthermore, their presence does not cause significant damage to the host [17,18]. Endophytic fungi are closely related to medicinal plants and promote the synthesis and accumulation of active ingredients in herbal medicines [19,20]. Some endophytic fungi can metabolize new natural products or produce the same or similar metabolites as the host plant [21]. Studies have shown that some endophytic fungi of Panax plants can produce ginsenosides, making endophytic fungi a new way to extract ginsenosides [22,23,24].

Studies have shown that ginsenosides have the effects of calming nerves, anticonvulsion, neuroprotection, improving intelligence, and preventing and treating AD [25]. AD is a neurodegenerative disease characterized by progressive memory loss and cognitive dysfunction. Senile plaque formed by β-amyloid protein (beta-amyloid protein, Aβ) deposition is one of its pathological markers [26]. The etiology and pathogenesis of AD have not been fully clarified, and the Aβ theory has been widely recognized [27]. The theory holds that Aβ is produced and aggregated excessively, which has neurotoxicity, causes neuron death, leads to memory loss and cognitive dysfunction, and finally causes AD. β-amyloid 42 (Aβ42) has higher self-aggregation and stronger neurotoxicity than other forms of Aβ and is the main factor causing AD. Under certain pathological conditions, β-secretase hydrolyzes the peptide bond of 670Met-671Asp to generate a transmembrane C segment with complete Aβ, which is passed through γ-secretase and further enzymatically cleaved to Aβ42 [28]. Therefore, an ideal inhibitor should have higher selectivity, mainly reduce the production of Aβ42, and also achieve the effect of inhibiting the production of Aβ42 by inhibiting the activity of β-secretase. Under the current trend of an increasingly aging population, the number of patients suffering from AD is also increasing. It is worth exploring whether ginsenosides have preventive and therapeutic effects on AD and the mechanism of action.

In this study, an endophytic fungus NSJG from wild ginseng was isolated and identified. This endophytic fungus was used to ferment the AGSL to explore whether the total saponin yield and ginsenoside types of fermented saponin extracts changed. Then, the fermented saponin extracts and relative rare ginsenosides were used to study their efficacy for AD by the molecular model of β-secretase and cell model of M146L cells. The research may improve the utilization value of AGSL and provide a reference for the prevention and treatment of AD.

## 2. Materials and Methods

### 2.1. Strains and Chemicals

The endophytic fungus NSJG used in this experiment was isolated from wild ginseng on Changbai Mountain, China.

Fungal Genomic DNA Extraction Kits, dimethyl sulfoxide, puromycin dihydrochloride, G418 antibiotics, and MTT were ordered from Solar bio Science & Technology Co. (Beijing, China); vanillin, absolute methanol, absolute ethanol, N-butanol, concentrated sulfuric acid, and chromatographically pure methanol were supplied by Shuang Shuang Chemical Co. (Yantai, China); twenty kinds of ginsenoside standards were bought from Master Biotechnology Co. (Chengdu, China); Dulbecco’s modified Eagle medium (DMEM)/high glucose and fetal bovine serum were purchased from Thermos Scientific (Shanghai, China); enhanced RIPA lysis buffer, human Aβ42 ELISA Kit, and Human β-Secretase ELISA Kit were ordered from Fantai Biotechnology Co. (Shanghai, China). β-Secretase Inhibitor HY-1675 was purchased from MCE (Shanghai, China).

The purity of vanillin is over 99.0%. The purity levels of absolute methanol, absolute ethanol, N-butanol, and chromatographically pure methanol are above 99.8%, 99.7%, 99.0%, and 99.9%, respectively. The purity of concentrated sulfuric acid is more than 98.0%. The purity of the 20 kinds of ginsenoside standards is more than 98.0%.

### 2.2. Identification of the Strain NSJG

The strain NSJG was identified through ITS sequence analysis. Template DNA was extracted following the protocol recommended by the Fungi Genomic DNA Extraction Kit. Endophytic fungi ITS sequences were PCR-amplified with universal primers (5′-TCCGTAGGTGAACCTGCGG-3′) and ITS4 (5′-TCCTCCGCTTATTGATATGC-3′). PCR products were electrophoresed on 1% agarose gel and sequenced at BGI (Beijing, China) Co., Ltd. Similarity of sequences was analyzed and determined using NCBI Blast (https://blast.ncbi.nlm.nih.gov/Blast.cgi, accessed on 27 September 2023) and EzTaxon (https://www.ezbiocloud.net/, accessed on 26 September 2023).

The phylogenetic analysis was conducted using MEGA [29] software version 5.0 with distance options according to the neighbor-joining method and was backed up by bootstrap values based on 1000 replicates [30].

### 2.3. Solid-State Fermentation of AGSL by Endophytic Fungus Strain NSJG

Four grams of AGSL powder and 36 mL of deionized water were added to a 100 mL conical flask as a solid-state fermentation medium. The solid medium was sterilized by an LDZX-50KBS vertical pressure steam sterilizer (Shenan, Shanghai, China) at 121 °C and 0.1 MPa for 30 min and inoculated endophytic fungus strain NSJG for solid fermentation.

Fermentation broth with NSJG mycelium pellets was prepared as liquid seeds. Four milliliters of liquid seeds were inoculated in a solid medium. The solid medium was fermented in a DH4000II constant temperature incubator (Tairuisi, Tianjin, China) at 26 °C. The fermented mixture was taken out until the mycelia were fully grown in the solid medium (about 16 d) and dried using a DHG-9070 electric blast dryer (Yuying, Shanghai, China) at 60 °C to constant weight for extraction of total saponins.

### 2.4. Extraction of Total Saponins

Four milliliters of anhydrous methanol was added to a reagent bottle, followed by 0.2 g of dried solid fermentation sample or unfermented AGSL. The bottle was ultrasonicated for 30 min at 500 W and 40 kHz in the KM-700DE ultrasonic instrument (Kunshanmeimei, Shanghai, China). The mixture was centrifuged at 10,000 rpm for 10 min in a KDC-160HR high-speed refrigerated centrifuge (Zhongjia, Anhui, China), resulting in a collection of supernatants. The supernatant was subjected to two rounds of precipitation and then dried in an RE-52AA rotary evaporation instrument (Xiande, Shanghai, China) at 40 °C. The extract was further extracted with 2 mL of water-saturated n-butanol three times and evaporated to dryness at 60 °C. The residue was dissolved in a small amount of methanol and diluted in a solution of 2 mL methanol. The solution was filtered into a brown reagent bottle to obtain total saponins and stored at −20 °C.

### 2.5. Determination of Total Saponins

Ultraviolet spectrophotometry was used for the determination of total saponins [22]. The color reaction of acid-hydrolysis products of sapogenins with vanillin was employed. The concentration of total saponins in the reaction sample was measured using a UV-2000 spectrophotometer (Unico, Shanghai, China) at a wavelength of 544 nm against a calibration curve established with an oleanolic acid standard. A total of 20 μL of total saponin solution was transferred to a test tube and allowed to evaporate. The residue was then dissolved in 5 mL of 72% sulfuric acid, followed by the addition of 0.5 mL of 8% vanillin ethanol. The test tubes were placed in an HH-2 constant temperature water bath pot (Huapuda, Jiangsu, China) at 60 °C for 10 min, followed by rapid cooling in an ice water bath for another 10 min. Absorbance was then measured at a wavelength of 544 nm, with the reaction solution without samples serving as a blank control. The concentration of total saponins was then calculated from the established standard curve.

### 2.6. Identification of Fermented Extracts

An LC/MS LTQ Orbitrap XL (Thermos Fisher Scientific, Shanghai, China) was used to analyze the saponin profile of fermented and unfermented AGSL. An Accucore C18 analytical column (2.6 μm, 150 mm × 2.1 mm) was used for saponin detection. Mobile phase A was deionized water and stationary phase B was acetonitrile. The programmed gradient was as follows: 0–13 min 77–54% A, 13–33 min 54–32% A, 33–45 min 32% A, 45–55 min 32–0% A, 55–60 min 0% A, 60–63 min 0–77% A. The flow rate was 0.3 mL/min and the injected volume was 10 μL. Detection was by absorbance at 203 nm and mass spectrum parameters were spray voltage 3.0 kv, sheath gas N_2_, and positive ion mode. The scanning range was M/Z 150–1300. A mixed standard solution consisting of pseudo-ginsenoside F11, noto-ginsenoside Fe, noto-ginsenoside Ft1, ginsenoside Rb1, Rb2, Rb3, Rc, Rd, Re, Rf, Rg1, Rg2, Rg3, Rh1, Rh2, Rk2, compound K (CK), F1, F2, and F3 at a concentration of 0.2 mg/mL was prepared for comparison and analysis with the database. The charge-to-mass ratio M/Z, peak time, and peak area of each substance were compared to determine the type and concentration of saponins [31].

### 2.7. Culture and Cell Viability Assay of M146L

The human APP-PS1 (M146L) cell line was obtained by transfecting the mutant PS1 gene into Chinese hamster ovary cells expressing the APP gene. M146L cells overexpress Aβ42. By measuring the concentration changes in Aβ42 secreted by M146L cells and the activity of β-secretase, it could be judged whether ginsenoside has an inhibitory effect on this process.

M146L cells were cultured in high-glucose DMEM containing 10% fetal bovine serum with G418 antibiotics (250 μg/mL) and puromycin (25 μg/mL) to maintain the expression of APP and PS1 genes. They were cultured in a constant temperature incubator at 37 °C with saturated humidity and 5% CO_2_ and sub-cultured every 2 d.

After M146L cells were cultured to the logarithmic growth phase, experimental and solvent control groups were set up for cell viability assay. The experimental group had different concentrations of fermented extracts or rare ginsenoside cells, and the solvent control group was 0.1%. Cell viability was measured using the MTT method [32]. Optical density (OD) was measured using a VDRTEX-5MK3 microplate reader (Redian, Shanghai, China) at 490 nm. The formula for calculating cell viability is as follows:

(1) Cell viability=experimental group OD/solvent control group OD

### 2.8. Measurement of Aβ42 Concentration and β-Secretase Activity by ELISA

After M146L cells were cultured to the logarithmic growth phase, different concentrations of fermented extracts or rare ginsenoside were added to the cells and cultured for 12 h, and the culture medium from each well was taken out for the measurement of Aβ42 concentration. The remaining adherent cells in the 96-well plate were lysed, and the lysate was taken out to measure β-secretase activity. The measurement procedures of Aβ42 concentration and β-secretase activity followed the protocols recommended by the Human Aβ42 ELISA Kit (Invitrogen, Waltham, MA, USA) and Human β-Secretase ELISA Kit (Invitrogen, Waltham, MA, USA).

### 2.9. Statistical Analysis

All experiments were replicated at least three times. Student’s *t*-test was employed for comparison between two groups, while a one-way analysis of variance was used for comparisons among multiple groups. Data were presented as means ± standard deviation (SD), *n* = 3. * *p* < 0.05, ** *p* < 0.01, *** *p* < 0.001.

## 3. Results

### 3.1. Identification of the Strain NSJG

**Figure 1:**
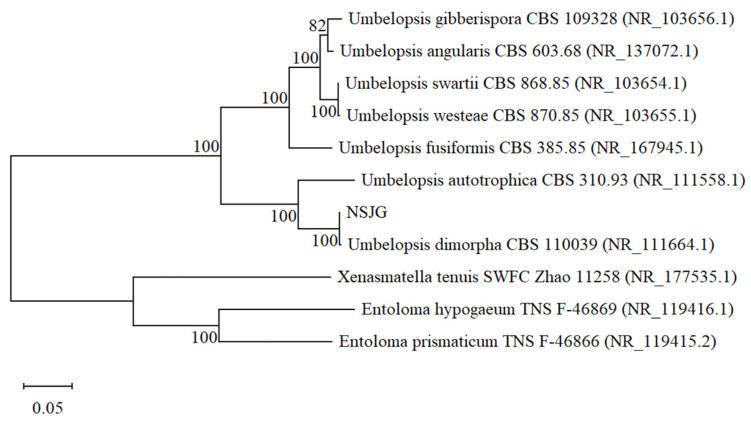
Phylogenetic tree characterization of endophytic fungus NSJG. The numbers on the nodes represent branch confidence values. The scale bar indicates a 2% estimated sequence divergence.

ITS sequencing showed that the highest sequence similarity of the strain NSJG was 100% with *Umbelopsis dimorpha* CBS 110039. As seen in Figure 1, the phylogenetic tree was constructed and showed that all branch confidences on the phylogenetic tree were greater than 80%. It indicated that each branch of this phylogenetic tree was relatively stable and could more accurately reflect the genetic evolutionary relationship between various species. The results demonstrated that the strain NSJG and *Umbelopsis dimorpha* CBS 110039 grouped with a high branch confidence (100%). Therefore, the strain NSJG was designated *Umbelopsis dimopha* (gene accession number: OR226416).

### 3.2. Determination of Total Saponin Concentration in Fermented Extracts of Biotransformed AGSL

**Figure 2:**
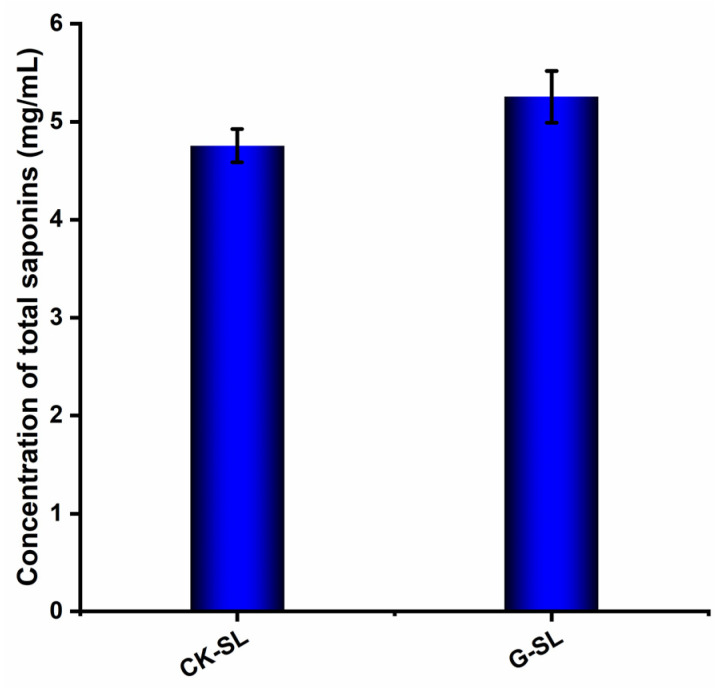
Total saponin concentration of CK-SL and G-SL. CK-SL represents a control group without fermentation. G-SL represents fermentation by NSJG and American ginseng stems and leaves. Data are presented as means ± SD, *n* = 3.

The oleanolic acid standard was prepared and detected for the drawing of the standard curve shown in Figure S1. As shown in Figure 2, the concentration of total saponins of biotransformed AGSL was calculated according to the standard curve by detecting the OD value at 544 nm. CK-SL was the control saponin extract of AGSL without fermentation. G-SL was the saponin extract of AGSL biotransformed by NSJG. The total saponin concentration of G-SL (5.254 ± 0.267 mg/mL) was higher than the control group CK-SL (4.755 ± 0.167 mg/mL). The results indicated that the total saponin concentration of AGSL was increased through fermentation by NSJG.

### 3.3. Identification and Analysis of Saponin Types in Fermented Extracts

The saponin types of standards, CK-SL, and G-SL were determined by LC/MS. All saponin standards can be detected within 30 min [33]. According to Figure S2 and Table 1, the extract of CK-SL contained 15 kinds of ginsenosides. Ginsenoside Rb1 level was the highest one. From Figure S3 and Table 2, the fermented extract of G-SL contained 13 kinds of ginsenosides. Compared with CK-SL, ginsenoside Rb1, Rb2, and Rg2 disappeared while a new ginsenoside, Rh1, was produced in G-SL. After AGSL was fermented by NSJG, the concentration of ginsenoside CK increased the most (561.79%), followed by ginsenoside Rh2 (77.48%), and ginsenoside F2 increased the least (40.89%). However, concentrations of the remaining seven ginsenosides and two noto-ginsenosides decreased. Among them, the decreasing proportion of seven ginsenosides from high to low was ginsenoside Rb3 (97.26%), ginsenoside Rd (87.14%), ginsenoside Rg1 (67.21%), ginsenoside Rg3 (65.57%), ginsenoside Rk2 (25.20%), ginsenoside F1 (23.53%), and ginsenoside Re (17.97%). The results indicated that ginsenosides Rd, Re, Rb1, Rb2, Rg1, Rg2, and Rg3 were biotransformed into rare ginsenosides Rh1, Rh2, CK, and F2.

**Figure 3:**
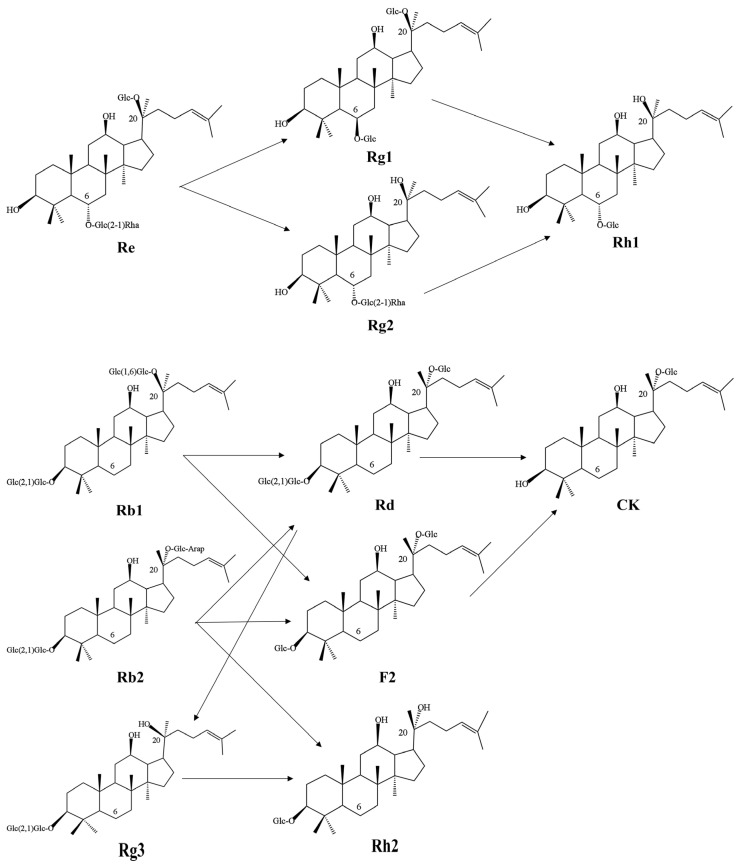
The transformation pathway of ginsenosides. Glc is β-D-glucopyranosyl; Arap is α-L-arabinopyranosyl; Rha is α-L-rhamnopyranosyl. Arrows represent the direction of ginsenoside transformation. The numbers represent the position of carbon in the chemical formula.

According to Table 1 and Table 2, rare ginsenoside Rh1 was newly produced after fermentation and concentrations of rare ginsenosides CK, Rh2, and F2 increased, with Rg2, Rb1, and Rb2 ginsenosides completely disappearing, and ginsenoside Re and Rg3 decreased significantly. The reason for the changes in the concentration and type of saponins was that glucose groups at the C3 and C20 positions of the ginsenosides of the protopanaxadiol saponin and the C6 and C20 positions of protopanaxatriol saponin were hydrolyzed during the fermentation process, resulting in rare ginsenosides. The possible conversion pathways are seen in Figure 3.

The transformation of ginsenoside Re into rare ginsenoside Rh1 can be achieved through two distinct pathways. The first involved the removal of a rhamnose molecule from the C-6 position to produce ginsenoside Rg1, followed by the removal of a glucose molecule from the C-20 position to generate rare ginsenoside Rh1. Alternatively, the glucose molecule was removed from the C-20 position initially to produce ginsenoside Rg2, then the rhamnose molecule was removed from the C-6 position, yielding rare ginsenoside Rh1.

Rare ginsenoside F2 was synthesized by removing glucose molecules from the C-3 and C-20 positions of ginsenoside Rb1 or by shedding glucose molecules from the C-3 position and arabinose molecules from the C-20 position of ginsenoside Rb2. Next, rare ginsenoside CK could be generated by the additional removal of a glucose molecule from the C-3 position of rare ginsenoside F2. Additionally, the intermediate product ginsenoside Rd was produced by eliminating either glucose or arabinose molecules from the C-20 position of ginsenosides Rb1 and Rb2. Finally, two glucose molecules were removed from the C-3 position of ginsenoside Rd to form rare ginsenoside CK.

In addition, ginsenoside Rd can produce ginsenoside Rg3 by removing a glucose molecule from the C-20 position, and then another glucose molecule was removed from the C-3 position of ginsenoside Rg3 to form rare ginsenoside Rh2. Interestingly, ginsenoside Rb2 could also directly produce rare ginsenoside Rh2 by removing glucose and arabinose molecules from the C-20 position and a glucose molecule from the C-3 position.

### 3.4. Cell Viability Assay of M146L

After the fermented extracts or ginsenosides are added to the cell culture medium, the culture time and concentration that are non-toxic to the cells should be selected first. Such conditions could ensure that the expression of Aβ42 in M146L cells is not affected by cytotoxicity. On this basis, the changes in Aβ42 concentration and β-secretase activity were caused by fermented extracts, which could indicate the preventive effect of saponin extracts on AD.

**Figure 4:**
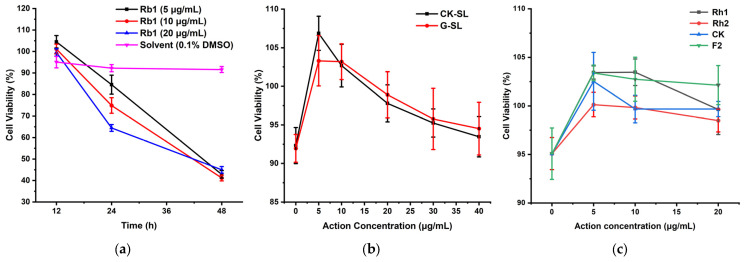
Cell viability at different times and concentrations. (**a**) Cell viability of ginsenoside Rb1 at 12, 24, and 48 h. (**b**) Cell viability of G-SL and CK-SL at different concentrations. (**c**) Cell viability of rare ginsenosides at different concentrations.

The ginsenoside Rb1 was selected to explore its effect on cell viability at three concentrations (5, 10, and 20 μg/mL) and three incubation times (12, 24, and 48 h). As shown in Figure 4a, cell viability in the 0.1% DMSO solvent control group did not change much and remained above 90% within 48 h. Cell viability of ginsenoside Rb1 was higher than that of the solvent control group after 12 h of culture and gradually decreased as the culture time increased. After 48 h of culture, cell viability was reduced to about 40%. The three action concentrations of ginsenoside Rb1 showed the same pattern. Therefore, a culture time of 12 h was chosen in a later experiment to ensure that cell viability would not be influenced. After 12 h of culture, ginsenoside Rb1 exhibited significantly different (*p* < 0.05) cell activity measurement results at 5 μg/mL compared to the solvent control group (0.1% DMSO). However, the differences in cell activity measurement at the other two concentrations were not significant.

The fermented extracts of G-SL and unfermented extracts of CK-SL with five concentrations (5, 10, 20, 30, and 40 μg/mL) were added to the medium of M146L cells to assay whether they were toxic after culture for 12 h. Figure 4b showed that the cell viability of CK-SL and G-SL at five concentrations was higher than that of the blank control. A comparison of the cell viability results of CK-SL at 5 and 10 μg/mL with the blank control demonstrated a significant difference (*p* < 0.05), and the difference at 20 μg/mL was even more significant (*p* < 0.01). Conversely, no significant difference was observed at 30 and 40 μg/mL. In addition, when G-SL and the blank control were compared, the only concentrations that resulted in a significant difference in cell activity were 5 and 10 μg/mL (*p* < 0.05). There was no significant difference in cell viability at the other three action concentrations.

As the rare ginsenosides of Rh1, Rh2, CK, and F2 were increased after fermentation, we also assayed whether these four rare ginsenosides with three concentrations (5, 10, and 20 μg/mL) had adverse effects on the cell viability. Figure 4c showed that they had no inhibitory effect on cell viability. Compared with the blank control, the rare ginsenoside Rh1 displayed a very significant difference in cell activity measurement results at 5 μg/mL (*p* < 0.01), with a significant difference at the concentration of 10 μg/mL (*p* < 0.05) while there was no significant difference at the action concentration of 20 μg/mL. The rare ginsenoside Rh2 revealed significant differences in cell activity measurement results at 5 and 10 μg/mL (*p* < 0.05) but not at 20 μg/mL. A non-significant difference was observed for the rare ginsenoside CK at 5 μg/mL, but significant differences were seen at 10 and 20 μg/mL (*p* < 0.05). Lastly, the rare ginsenoside F2 displayed an extremely significant difference in cell activity measurement results at the concentration of 5 μg/mL (*p* < 0.01), with significant differences also observed at 10 and 20 μg/mL (*p* < 0.05).

The above results indicated that fermented extracts of G-SL, unfermented extracts of CK-SL, and four kinds of rare ginsenosides did not cause toxicity to the cells, and the subsequent determination of Aβ42 concentration and β-secretase activity could be carried out.

### 3.5. Evaluation of the Preventive Effect of Fermented Extracts on AD by the Changes in Aβ42 Concentration and β-Secretase Activity

**Figure 5:**
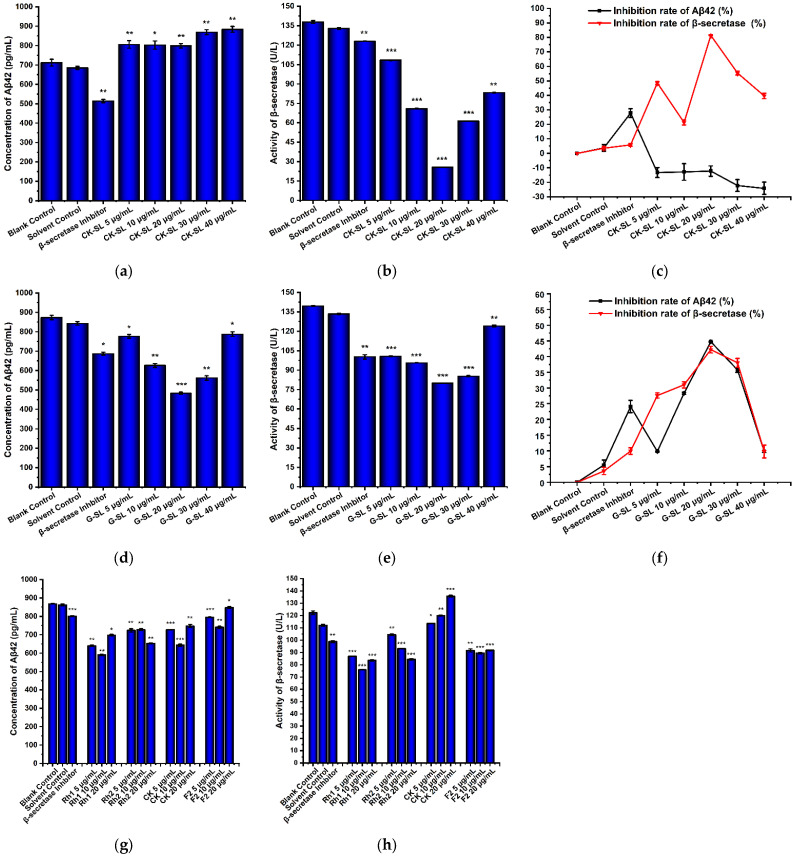
Effects on Aβ42 concentration and β-secretase activity by fermented extracts and rare ginsenosides. (**a**) Aβ42 concentration of CK-SL at different concentrations. (**b**) β-secretase activity of CK-SL at different concentrations. (**c**) The inhibition rate of Aβ42 and β-secretase by CK-SL at different concentrations. (**d**) Aβ42 concentration of G-SL at different concentrations. (**e**) β-secretase activity of G-SL at different concentrations. (**f**) The inhibition rate of Aβ42 and β-secretase by G-SL at different concentrations. (**g**) Aβ42 concentration by four rare ginsenosides. (**h**) β-secretase activity by four rare ginsenosides. Data are presented as means ± SD, *n* = 3. * *p* < 0.05, ** *p* < 0.01, *** *p* < 0.001.

First, standard curves of Aβ42 and β-secretase activity were detected and drawn with the concentration of the standard substance as the abscissa and the OD value as the ordinate (Figures S4 and S5). The experimental groups (5, 10, 20, 30, and 40 μg/mL of CK-SL and G-SL), a blank control group (DMEM), a solvent control group (0.1% DMSO), and a positive control group (β-secretase inhibitor) were set up and cultured for 12 h, and Aβ42 concentration and β-secretase activity were measured. Compared with the solvent control group, CK-SL of five concentrations had no inhibitory effect on the Aβ42 concentration (Figure 5a). However, they all inhibited the β-secretase activity (Figure 5b). CK-SL had a very significant inhibitory effect on the β-secretase activity at 40 μg/mL and a more significant inhibitory effect at 5, 10, 20, and 30 μg/mL. Compared with the solvent control group, G-SL of five concentrations inhibited the Aβ42 concentration and the β-secretase activity. They had a significant inhibitory effect on the Aβ42 concentration. G-SL had a very significant inhibitory effect at 10 and 30 μg/mL and a more significant inhibitory effect at 20 μg/mL (Figure 5d). G-SL had a very significant inhibitory effect on β-secretase activity at 40 μg/mL and a more significant inhibitory effect at 5, 10, 20, and 30 μg/mL (Figure 5e).

Excessive accumulation of Aβ42 is the main cause of AD. There are multiple mechanisms leading to increased accumulation of Aβ42, the main one being the β-secretase hydrolysis pathway, in which β-secretase hydrolyzes Aβ protease to generate Aβ42. Therefore, this study focused on substances that inhibit both Aβ42 biosynthesis and β-secretase activity. Only by simultaneously reducing intracellular β-secretase activity and Aβ42 concentration can the β-secretase hydrolysis pathway be better inhibited, thereby achieving a better therapeutic effect on AD. Analysis of the overall change trend of inhibition rate of Aβ42 concentration and β-secretase activity indicated that CK-SL could only inhibit β-secretase activity but not Aβ42 concentration (Figure 5c), while G-SL inhibited both β-secretase activity and Aβ42 concentration (Figure 5f). The optimal inhibitory concentration of G-SL was 20 μg/mL, and its inhibition rates of Aβ42 concentration and β-secretase activity were 42.75% and 42.22%. Therefore, the inhibition rate of AD by G-SL was better than CK-SL.

Next, considering that rare ginsenosides Rh1, Rh2, CK, and F2 were increased in the fermented extract G-SL, these four rare ginsenosides at 5, 10, and 20 μg/mL were used to examine their impact on the Aβ42 concentration and β-secretase activity. We aimed to investigate the mechanisms underlying the preventive and therapeutic effects of fermented extracts in AD. Compared with the solvent control group, the four rare ginsenosides all had inhibitory effects on the Aβ42 concentration (Figure 5g), with the following rank of Rh1 with the highest inhibition rate of 31.41% at 10 μg/mL, Rh2 with the highest inhibition rate of 24.18% at 20 μg/mL, CK with the highest inhibition rate of 25.35% at 10 μg/mL, and F2 with the highest inhibition rate of 14.00% at 10 μg/mL. Rare ginsenosides Rh1, Rh2, and F2 had inhibitory effects on β-secretase activity except for rare ginsenoside CK (Figure 5h). Also, rare ginsenoside Rh1 had the best inhibitory effect on the activity of β-secretase. The inhibitory effect is more significant at 10 μg/mL, and the inhibition rate (32.29%) was about three times higher than that of the β-secretase inhibitor (11.86%). In short, rare ginsenoside Rh1, Rh2, and F2 had a better inhibitory effect on the β-secretase hydrolysis pathway than CK.

## 4. Discussion

In this study, an endophytic fungal strain NSJG isolated from wild ginseng in China was identified as *Umbelopsis dimopha*. The strain NSJG could directly ferment AGSL in a solid state, increase total saponin concentration, and transform some common ginsenosides into rare ginsenosides. To our knowledge, this is the first report that the endophytic *Umbelopsis dimopha* successfully transformed saponins in AGSL into rare ginsenosides through solid-state fermentation. Previous research concerning the transformation of ginsenoside mostly focused on the conversion of one or a few monomers. For example, *Aspergillus niger* strain XD101 isolated from Panax noto-ginseng could transform ginsenoside Rb1 into CK [34]. Ginsenoside Rb1 could also be transformed into ginsenoside CK by the endophytic fungus *Arthrinium* sp. GE 17–18 isolated from Panax ginseng [35]. *Aspergillus niger* strain TH-10a and *Paecilomyces bainier* strain 229-7 could convert ginsenoside Rb1 to Rd [36,37,38]. In this experiment, AGSL was directly fermented by endophytic fungus *Umbelopsis dimopha* strain NSJG isolated from wild ginseng. The results suggested that four kinds of rare ginsenosides, Rh1, Rh2, F2, and CK, could be successfully obtained by the transformation of some common ginsenosides that existed in AGSL. This research improved the utilization value of stems and leaves.

Although there are many kinds of ginsenosides, they all contain similar structures. The saponins in AGSL are mainly dammarane, including panaxadiol and panaxatriol types [39]. In this study, rare ginsenoside Rh1 was newly produced and the yield of rare ginsenosides Rh2, F2, and CK was increased in the fermented extract of AGSL by NSJG. The yield and type of ginsenosides changed mainly because glucose groups at the C-3 and C-20 positions of panaxadiol type ginsenosides or C-6 and C-20 positions of panaxatriol type ginsenosides were hydrolyzed, thereby obtaining rare ginsenosides. The process of using microorganisms to transform ginsenosides into rare ginsenosides with higher medicinal value was safe and non-toxic, reduced the generation of by-products, and saved the cost of separating ginsenosides from total saponins [40,41].

β-secretase is the key enzyme of the Aβ pathway under the pathological conditions that cause AD [42]. It is a new type of aspartic acid protease fixed to the membrane, which is called BACE [43]. The BACE gene is located on chromosome 11, encodes a 501 amino acid sequence, and contains two active centers DTGS and DSGT, and mutations in either center will lose the catalytic activity of this enzyme [44]. Therefore, inhibiting the expression of the BACE gene can prevent the Aβ pathway, thereby achieving the therapeutic effect of AD [45]. Currently, there are many types of compounds used to study the inhibition of β-secretase activity, most of which are peptide inhibitors, but there are fewer studies on triterpenoid inhibitors. It has been reported that glycosaminoglycan extract composed predominantly of 4-sulfated chondroitin sulfate possessed the ability to inhibit BACE [46]. 3-Hydroxyhericenone F from *Hericium erinaceus* was a promising naturally occurring chemical constituent for AD via the inhibition of the β-secretase enzyme [47]. Interestingly, the fermented extract G-SL from solid-state fermentation of AGSL by endophytic fungus NSJG could inhibit the activity of β-secretase and reduce the concentration of Aβ42, thereby inhibiting the β-secretase hydrolysis pathway under pathological conditions. However, the unfermented extract CK-SL could only reduce the β-secretase activity but not the Aβ42 concentration. Therefore, the fermented extract of AGSL exhibits stronger inhibitory effects on Alzheimer’s disease. This extract undergoes fermentation to produce rare ginsenoside Rh1, as well as an increased yield of Rh2, F2, and CK. Some research has elucidated the profound effect of the rare ginsenoside Rh2 in enhancing cholinergic transmission, suppressing oxidative stress, and fostering synaptic plasticity, thereby mitigating memory dysfunction, particularly spatial memory linked to the hippocampus [48]. Additionally, the rare ginsenoside F2 has been found to exhibit promising efficacy in AD treatment by inhibiting acetylcholinesterase activity and diminishing Aβ deposition [49]. Furthermore, the ginsenoside CK has been demonstrated to exert beneficial effects on memory function by modulating Aβ aggregation and promoting the transduction of the Nrf2/Keap1 signaling pathway, ultimately leading to a reduction in oxidative damage to neurons and inhibition of neuronal apoptosis [50]. However, there are no existing studies investigating the potential benefits of rare ginsenoside Rh1 in the treatment of AD. This study proved these four rare ginsenosides have strong inhibitory effects on AD, of which Rh1 showed over three times higher inhibition than a β-secretase inhibitor. The results revealed the underlying mechanism for the better inhibitory effect of the fermented extract. However, the mechanism by which G-SL can act as a β-secretase inhibitor is not yet specific. Further research is needed to explore whether ginsenosides affect the expression of the BACE gene.

## 5. Conclusions

The endophytic fungus NSJG isolated from wild ginseng was identified as *Umbelopsis dimorpha*. The yield of total saponins in AGSL fermented by NSJG was increased. Also, some ginsenosides were transformed into rare ginsenosides. It improved the utilization value of AGSL. The extracts from fermented AGSL had a good effect on AD by inhibiting the concentration of Aβ42 and the activity of β-secretase. Therefore, the endophytic fungus NSJG and its transformed AGSL have very high application value in saponin acquisition and AD prevention.

## Figures and Tables

**Table 1:** Results of LC/MS analysis of CK-SL fermented extracts.

Ginsenoside Type	RT (min)	M/Z (M + Na)	Calculated Mass Errors (ppm)	Area	Concentration (mg/L)
Ginsenoside Rg1	4.64	823.4784	3.04	85,245,657	40.47 ± 1.22
Ginsenoside Re	4.84	969.5361	2.78	152,532,379	82.05 ± 1.11
Pseudo-ginsenoside F11	8.88	823.4778	3.76	178,134,984	54.54 ± 1.50
Ginsenoside Rb1	10.06	1131.5886	−1.06	58,056,561	730.79 ± 4.20
Ginsenoside Rg2	10.73	807.4833	3.22	41,858,831	106.53 ± 1.29
Ginsenoside Rb2	11.01	1101.5778	2.90	49,691,314	22.26 ± 1.16
Ginsenoside Rb3	11.13	1101.5776	3.09	60,010,017	31.61 ± 1.24
Ginsenoside F1	11.55	661.4265	2.27	21,308,120	6.79 ± 0.91
Ginsenoside Rd	11.96	969.5357	0.41	119,144,652	75.29 ± 0.16
Noto-ginsenoside Fe	14.03	939.5225	6.07	91,980,256	57.52 ± 2.43
Ginsenoside F2	15.54	807.4828	3.84	84,843,618	45.81 ± 1.54
Ginsenoside Rg3	17.54	807.4832	3.34	76,407,798	40.90 ± 1.34
Ginsenoside CK	22.08	645.4313	2.79	61,108,452	13.09 ± 1.12
Ginsenoside Rh2	23.80	645.4312	2.94	74,645,154	64.44 ± 1.18
Ginsenoside Rk2	30.57	627.4207	3.03	15,798,842	24.21 ± 1.21

**Table 2:** Results of LC/MS analysis of G-SL fermented extracts.

Ginsenoside Type	RT (min)	M/Z (M + Na)	Calculated Mass Errors (ppm)	Area	Concentration (mg/L)
Ginsenoside Rg1	4.70	823.4783	3.16	27,951,782	13.27 ± 1.26
Ginsenoside Re	4.80	969.5358	3.09	125,128,845	67.31 ± 1.24
Pseudo-ginsenoside F11	8.87	823.4782	3.28	147,836,511	45.27 ± 1.31
Ginsenoside Rh1	10.95	661.4263	2.57	18,065,779	53.62 ± 1.03
Ginsenoside Rb3	11.12	1101.5776	3.09	1,642,635	0.87 ± 0.12
Ginsenoside F1	11.50	661.4262	2.72	16,295,188	5.20 ± 0.11
Ginsenoside Rd	12.02	969.5361	0.00	15,323,684	9.68 ± 1.00
Noto-ginsenoside Fe	13.47	939.5253	3.09	14,396,025	9.00 ± 1.24
Ginsenoside F2	15.56	807.4832	3.34	119,537,757	64.54 ± 1.34
Ginsenoside Rg3	17.59	807.4836	2.85	26,305,526	14.08 ± 1.14
Ginsenoside CK	22.05	645.4315	2.48	404,408,782	86.65 ± 0.99
Ginsenoside Rh2	23.74	645.4315	2.48	132,476,841	114.37 ± 0.99
Ginsenoside Rk2	30.53	627.4208	2.87	11,817,115	18.11 ± 1.15

## Data Availability

The data presented in this study are available on request from the corresponding author.

## Supplementary Materials

The following supporting information can be downloaded at: https://www.mdpi.com/article/10.3390/nu15234878/s1, Figure S1: Standard curve for concentration of total saponin; Figure S2: TIC of LC/MS determination by CK-SL; Figure S3: TIC of LC/MS determination by G-SL; Figure S4: Standard curve for concentration of Aβ42; Figure S5: Standard curve for concentration of β-secretase.
