# Supplementary material for: Biotransformation of American Ginseng Stems and Leaves by an Endophytic Fungus Umbelopsis sp. and Its Effect on Alzheimer’s Disease Control
Source: Nutrients. 2023 Nov 22;15(23):4878. doi: 10.3390/nu15234878 (PMC10708258; doi:10.3390/nu15234878)
Supplement: Supplementary file 1 [file nutrients-15-04878-s001.zip › nutrients-2715973-supplementary.pdf]

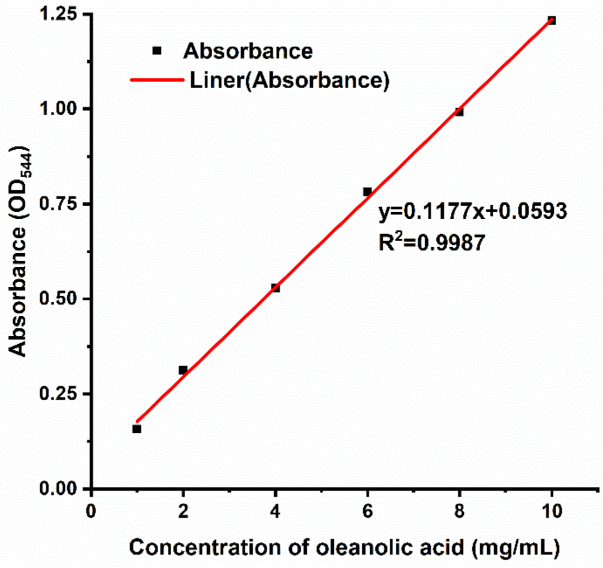

Figure S1. Standard curve for concentration of total saponin.

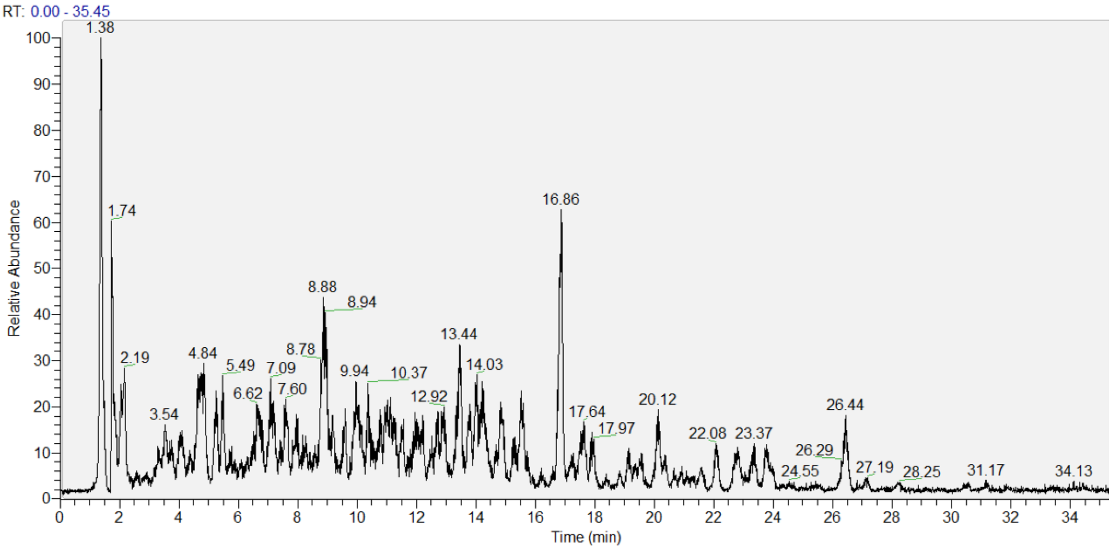

Figure S2. TIC of LC/MS determination by CK-SL.

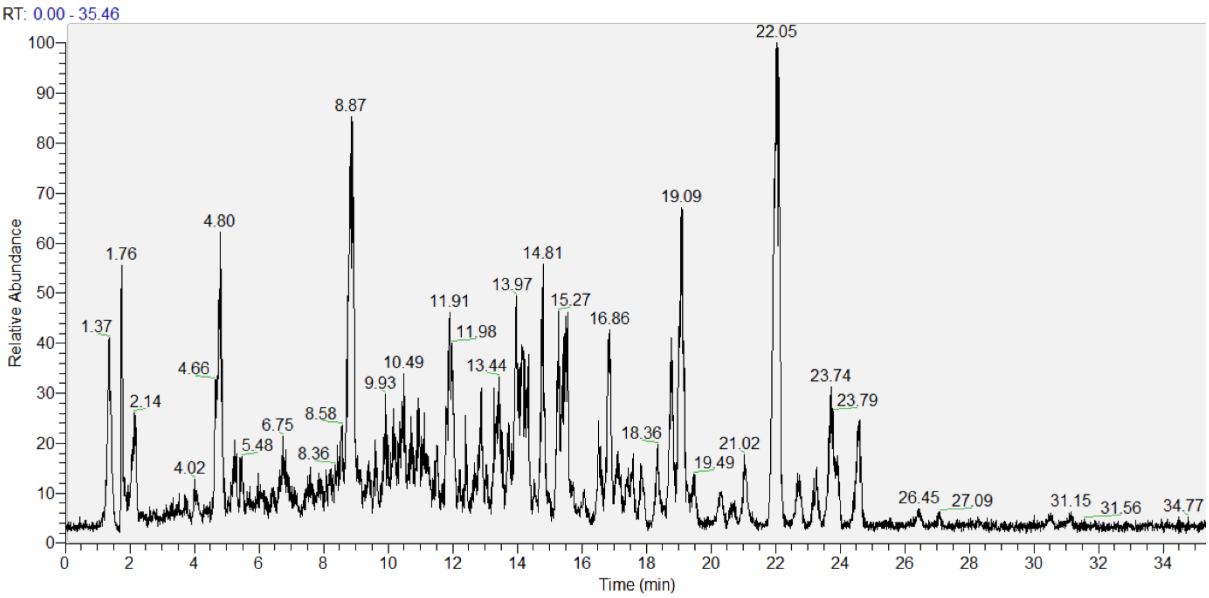

Figure S3. TIC of LC/MS determination by G-SL.

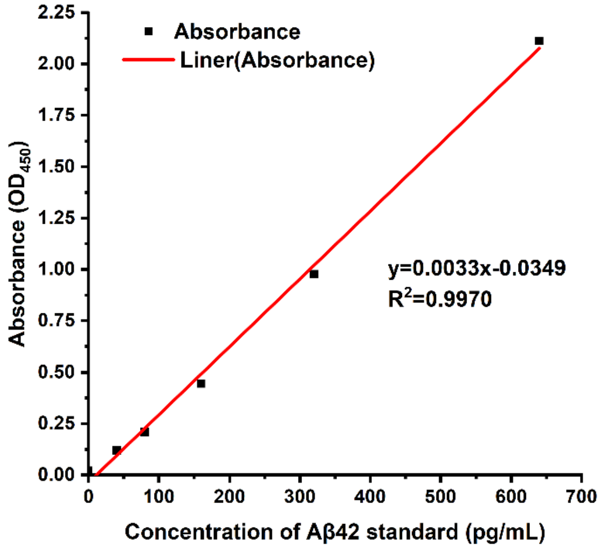

Figure S4. Standard curve for concentration of Aβ42.

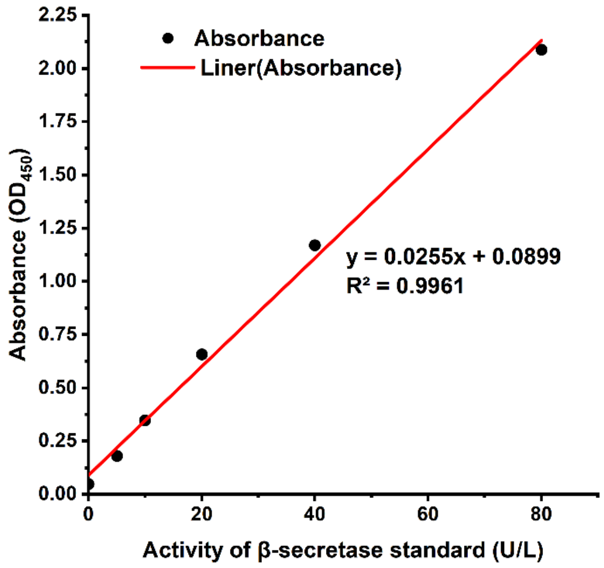

Figure S5. Standard curve for activity of β-secretas.
